# Improving the Thermostability and Activity of Transaminase From *Aspergillus terreus* by Charge-Charge Interaction

**DOI:** 10.3389/fchem.2021.664156

**Published:** 2021-04-14

**Authors:** Jia-Ren Cao, Fang-Fang Fan, Chang-Jiang Lv, Hong-Peng Wang, Ye Li, Sheng Hu, Wei-Rui Zhao, Hai-Bin Chen, Jun Huang, Le-He Mei

**Affiliations:** ^1^School of Biological and Chemical Engineering, Zhejiang University of Science and Technology, Hangzhou, China; ^2^School of Biotechnology and Chemical Engineering, NingboTech University, Ningbo, China; ^3^Enzymaster (Ningbo) Bio-Engineering Co., Ltd., Ningbo, China; ^4^Jinhua Advanced Research Institute, Jinhua, China; ^5^Department of Chemical and Biological Engineering, Zhejiang University, Hangzhou, China

**Keywords:** amine transaminase, thermostability, enzyme thermal stability system, site-directed mutagenesis, molecular dynamics simulations

## Abstract

Transaminases that promote the amination of ketones into amines are an emerging class of biocatalysts for preparing a series of drugs and their intermediates. One of the main limitations of (*R*)-selective amine transaminase from *Aspergillus terreus* (*At*-ATA) is its weak thermostability, with a half-life (*t*_1/2_) of only 6.9 min at 40°C. To improve its thermostability, four important residue sites (E133, D224, E253, and E262) located on the surface of *At*-ATA were identified using the enzyme thermal stability system (ETSS). Subsequently, 13 mutants (E133A, E133H, E133K, E133R, E133Q, D224A, D224H, D224K, D224R, E253A, E253H, E253K, and E262A) were constructed by site-directed mutagenesis according to the principle of turning the residues into opposite charged ones. Among them, three substitutions, E133Q, D224K, and E253A, displayed higher thermal stability than the wild-type enzyme. Molecular dynamics simulations indicated that these three mutations limited the random vibration amplitude in the two α-helix regions of 130–135 and 148–158, thereby increasing the rigidity of the protein. Compared to the wild-type, the best mutant, D224K, showed improved thermostability with a 4.23-fold increase in *t*_1/2_ at 40°C, and 6.08°C increase in $T_{50}^{10}$. Exploring the three-dimensional structure of D224K at the atomic level, three strong hydrogen bonds were added to form a special “claw structure” of the α-helix 8, and the residues located at 151–156 also stabilized the α-helix 9 by interacting with each other alternately.

## Introduction

Chiral amines are important components of many significant bioactive compounds, pharmaceutical intermediates and agrochemical industry products (Bornscheuer et al., 2012; Mathew and Yun, 2012a; Ghislieri and Turner, 2014; Park et al., 2014; Fuchs et al., 2015; Ferrandi and Monti, 2017; Dawood et al., 2018; Cai et al., 2020). In addition to optical rotation, enantiomers of chiral drugs have the same physical properties, but they are absorbed, activated or degraded by the metabolic system of the human body in different ways during the pharmacological action, resulting in different efficacies and toxicities (Burke and Henderson, 2002). Because of their broad-spectrum biological activities and high purity in the synthesis of enantiomeric amines, they can act as chiral building blocks for the synthesis of more complex structural drugs (Breuer et al., 2004; Nugent, 2010). For instance, aromatic chiral amine derivatives are intermediates of the highly potent KCNQ2 opener (Wu et al., 2004), rivastigmine agents to treat Alzheimer's disease (Hua et al., 2018), and anti-arthritic drugs (Dyckman et al., 2011). Unfortunately, synthetic routes for these compounds are still challenging.

Many enzymes have been employed for the synthesis of chiral amines, including transaminases, imine reductases, amine dehydrogenases, and reductive aminases (Turner and Truppo, 2010; Łyskowski et al., 2014; Godwin et al., 2017; Gomm and O'Reilly, 2018; Jiang et al., 2020). Among them, the coenzyme PLP of transaminase can be recycled, unlike many expensive coenzymes, which gradually deplete the amino products synthesized *in situ* (Paul et al., 2014). In particular, amine transaminases (ATAs) have many industrial advantages and chemical properties over conventional chemical synthesis of optically pure chiral amines, including excellent stereoselectivity (Svedendahl et al., 2010) and broad substrate spectrum (Guo and Berglund, 2016). More importantly, ATAs can achieve continuous flow biotransformations under mild conditions (Andrade et al., 2014) as an alternative technology to replace toxic, non-recyclable chemical catalysts and reduce the use of high- temperature and high-pressure conditions in the chemical production process.

Transaminases have been extensively studied during the past few years for the synthesis of chemically pure chiral amines (Hhne and Bornscheuer, 2009; Zhu and Hua, 2009; Hailes et al., 2010; Nugent, 2010; Tufvesson et al., 2011; Malik et al., 2012; Mathew and Yun, 2012b; Kroutil et al., 2013). However, transaminases usually require better reaction rates, higher temperature adaptability in industrial production, and reduce risk of microbial contamination. Protein engineering plays a vital role in enhancing the thermal stability of (*R*)-selective *At*-ATA to expand its applicability in industrial processes (Liu et al., 2019). To date, the rational design of protein engineering involves many factors, such as surface electrostatic interactions, hydrophobic interactions, B-factor values, consensus mutagenesis, disulphide bridges, coevolution networks, and hydrogen bonding interactions (Pace et al., 2011; Wang et al., 2014; Zhang et al., 2014, 2020; Huang et al., 2017; Xie et al., 2018, 2019; Moon et al., 2019; Zhu et al., 2019; Cao et al., 2020). All of these have been employed to develop stable proteins.

Recently, the enzyme thermal stability system (ETSS), a suite of computational programs based on TK-SA model calculation and surface charge-charge interaction analysis was released (Zhang et al., 2014). The TK model was constructed by Tanford and Kirkwood in 1957 (Tanford and Kirkwood, 1957), based on the charge-charge interaction to describe the electrostatic properties of the whole protein (Matthew et al., 1985; Matthew and Gurd, 1986a,b). After solvent accessibility (SA) was introduced to refine the TK-SA model, Bashford and Karplus majorized the TK-SA model with the effects of partition function (Sanchez-Ruiz et al., 1999) and Gibbs free energy (Matthew et al., 1979; Richmond, 1984; Bashford and Karplus, 1991), which has been successfully applied in protein modification engineering (Elcock, 2001; Sanchez-Ruiz and Makhatadze, 2001; Ibarra-Molero and Sanchez-Ruiz, 2002; Makhatadze et al., 2003, 2004; Strickler et al., 2006; Gribenko et al., 2009; Schweiker and Makhatadze, 2009).

In this work, the potential mutation sites were replaced with electrically neutral amino acids on the protein surface based on the TK-SA model, which was used to construct the mutant with enhanced thermostability. Subsequently, the prospective stabilizing effects of these mutations were verified by thermal inactivation experiments, and basic-amino-acid scanning was more accurate in finding the most thermal-stability-improved mutation at each site. In addition, these variants at each site were analyzed by molecular dynamics (MD) simulation (Purmonen et al., 2007), with the aim of exploring the improved thermostability and catalytic activity of the mutants at the atomic level.

## Materials and Methods

### Materials

The *At*-ATA cDNA from *Aspergillus terreus* sequence, including the *Nco*I and *Xho*I restriction sites, was synthesized by General Biosystems (Chuzhou, China), and the plasmid pET-28a(+) was used for gene cloning and DNA sequencing. All PCR primers were synthesized by Qingke Biology Co., Ltd. (Hangzhou, China). PrimeSTAR^®^ Max DNA polymerase was obtained from Takara Biotechnology (Dalian, China) for the polymerase chain reaction (PCR). *Dpn* I, Yeast extract and tryptone were obtained from Thermo Fisher Scientific (Shanghai, China). Dimethyl sulfoxide (DMSO), 1-(*R*)-PEA and pyruvate were obtained from Aladdin Biochemical Technology Co., Ltd. (Shanghai, China). NaCl, NaH_2_PO_4_, Na_2_HPO_4_, NaOH, DNA ladder, protein marker, protein loading buffer, kanamycin sulfate, isopropyl-β-d-thiogalactoside (IPTG), Ni-NTA Sefinose (TM) Resin (Settled Resin) kit, SDS-PAGE gel kit, and Modified Bradford Protein Assay Kit were obtained from Sangon (Shanghai, China). *E. coli* BL21(DE3) Chemically Competent Cell, EasyPure^®^ HiPure Plasmid MaxiPrep Kit, EasyPure^®^ Quick Gel Extraction Kit and EasyPure^®^ PCR Purification Kit were purchased from TransGen Biotech (Beijing, China).

### Location of the Mutant Sites

Based on the crystal structure of *At*-ATA (PDB ID: 4CE5) obtained from the Protein Data Bank (http://www.rcsb.org), ETSS was used to calculate the total interaction energy between charged amino acids at points *i* and *j* (*E*_*ij*_) from wild-type *At*-ATA. Based on these results, we selected the modification of residues with positive *E*_*ij*_ values, which signified an unfavorable interaction, to enhance the *At*-ATA thermostability. In addition, three principals were used to redesign *At*-ATA mutants: (i) residues with high positive *E*_*ij*_ values were priorities, (ii) residues predicted to form hydrogen bonds with the nearby conserved in *At*-ATA, and (iii) residues far from the catalytic and binding pocket were selected to retain the activity. When negatively charged amino acids are involved in the mutation residues, it is a good strategy to turn the target to be amidated to approach electric neutrality. When both negatively and positively charged residues located at the same position were failed to depress the *E*_*ij*_ values or turned to the opposite ones, the charged residues will attempt to be alanine. All three-dimensional (3D) structures of *At*-ATA were visualized using PyMOL 2.0.7 software (http://pymol.org).

### Site-Directed Mutagenesis

The *At*-ATA gene from *A. terreus* was cloned into pET-28a(+), using *Nco* I and *Xho* I, and transformed into *E. coli* DH5a. All primers used for site-directed mutagenesis are listed in Supplementary Table 1. For the mutagenic PCR, first stage: 98°C for 1 min, one cycle; second stage: 98°C, 15 s/55°C, 15 s/72°C, 2 min, 30 cycles; and third stage: 72°C for 10 min, one cycle; 2 × PrimeSTAR^®^ Max DNA polymerase (1 ×, 25 μL), forward and reverse primers (10 μM, 1 μL each) together with template (pET-28a(+)-*At*-ATA; 0.1 ng/μL, 2 μL), and diluted with ddH_2_O to 50 μL. The PCR products were purified using an EasyPure^®^ PCR Purification Kit. Following depuration, *Dpn* I (20 U) was added to the buffer, and the mixture was incubated for 3 h at 37°C and transformed into *E. coli* BL21 (DE3) bacteria by heat shock. After screening on a Luria-Bertani (LB) plate containing 50 μg/mL kanamycin and incubated at 37°C overnight, the sequence of the mutated plasmid DNA was aligned and checked.

### Protein Expression and Purification

The colony was designed by transferring the recombinant plasmid into *E. coli* BL21 (DE3) using the EasyPure^®^ HiPure Plasmid MaxiPrep Kit. Protein was expressed by adding a colony to LB-Kan medium (5 mL; 50 μg/mL), and the strain was grown at 37°C and 200 rpm for 6 h, as the OD_600_ reached ~0.6. The culture was then added to LB-Kan medium (200 mL; 50 μg/mL) and allowed to continue growing at 37°C and 180 rpm for another 3 h. Thereafter, the protein expression was induced by adding IPTG to a final concentration of 1 mM and returned to 25°C, 150 rpm, for 20 h. Subsequently, the cells were centrifuged at 4°C (6,000 × g, 6 min), then washed twice with buffer A (50 mM sodium phosphate buffer, and pH 8.0). The cells were later dissolved in 55 mL of buffer B (300 mM NaCl, 50 mM sodium phosphate buffer, 20 mM imidazole, and pH 8.0) and disrupted using a high-pressure homogenizer (ATS, Jiangsu, People's Republic of China) for 1 min in 140 mPa until the mixture became clear. After the protein solution was centrifuged at 8,000 rpm for 55 min at 4°C, the purified protein, containing an *N*-terminal His_6_-tag, was attained using a Ni-NTA Sefinose column and eluted with buffer C (300 mM NaCl, 50 mM sodium phosphate buffer, 250 mM imidazole, and pH 8.0). Furthermore, SDS-PAGE (12% separating and 5% stacking gels) and a Modified Bradford Protein Assay Kit (Sangon Biotech Co., Ltd. Shanghai, China) were used to analyze purified proteins. Protein concentrations were determined by the Bradford method using BSA as a standard.

### Thermostability of the *At*-ATA and Variants

Both the half-life (*t*_1/2_) and half-life temperature ($T_{50}^{10}$) values characterize the thermostability of the enzyme. *t*_1/2_ is defined as the time when the residual activity of *At*-ATA and its mutants was reduced to 50% of its original activity at 40°C. Similarly, $T_{50}^{10}$ refers to the temperature at which the enzyme activity was reduced to half of the original activity after heat treatment at a continuous temperature for 10 min.

The purified *At*-ATA and its mutants were incubated for 0–30 min at 40°C, and then cooled on ice for 10 min. Enzyme activity test was performed at 25°C for 3 min. An exponential function model: Exp2PMod1 [formula: y = exp(-*k*_*d*_·t)] by nonlinear regression was used to fit our data using Origin 8.0, then the first-order rate constants (*k*_*d*_) and 50% of relative enzyme activity were determined. In addition, the enzyme solution was incubated for 10 min at temperatures of 4, 25, 35, 37, 40, 42, 45, 50, and 55°C, and then cooled on ice for 10 min. The data were fitted to a four-parameter Boltzmann sigmoidal function reformed with the Levenberg-Marquardt iterative algorithm. The formula is presented in Equation (1).

(1) $$\begin{array}{r} R=A+\frac{B-A}{1+e^{\frac{\left(T_{m}-T\right)}{C}}} \end{array}$$

Where *R* is the percentage of residual activity at temperature *T, A*, and *B* are the pretransitional and posttransitional percentages of residual activity, respectively, and C is the slope factor. In the absence of treatment, *T*_m_ = *T*_0._

Differential scanning fluorimetry (DSF) is a rapid and highly efficient method for identifying protein thermostability (Niesen et al., 2007). The protein unfolding temperature was gauged by increased fluorescence of the dye molecules. The dye molecules have affinity for hydrophobic portions of the protein, exposed as the protein unfolds. The mixture consisted of 1 mg/mL pure enzyme, 1× SYPRO Orange dye (dissolved in DMSO) and diluted with buffer C (150 mM NaCl, 50 mM sodium phosphate buffer, and pH 8.0) to 50 μL. The sample with buffer C instead of pure enzyme was used as the negative control. The measurements were performed on a StepOne Real-Time PCR System (Applied Biosystems, USA). The temperature from 25 to 70°C was scanned in 0.7°C increments, with each temperature maintained for 30 s. The excitation and emission wavelengths were 490 and 605 nm, respectively. The melting temperature *T*_m_ was calculated using the formula shown in Equation (2):

(2) $$\begin{array}{r} y=UF+\;\frac{NF-UF}{1+e\frac{Tm-x\;}{\alpha }} \end{array}$$

Where *UF* and *NF* are the minimum and maximum emission fluorescence intensities, respectively, and α is the slope of the curve within *T*_m_.

### Enzyme Activity Assay and Kinetic Parameters

The activities of the wild-type and mutant strains were measured as described by Schätzle et al. (2009). One unit of activity was defined as the amount of enzyme required to release 1 μmol of acetophenone per min under the assay conditions. The substrate pre-mixture was prepared containing 2.5 mM 1-(*R*)-PEA, and 2.5 mM pyruvate, 0.1 mM PLP and 0.25% (w/v) DMSO in 180 μL of buffer A, and 20 μL of enzyme concentrated with buffer A. The reaction was measured in UV 96-well microtiter plates at 25°C and 245 nm for 3 min, and the production of acetophenone was monitored using an MD 190 photometer (Molecular Devices, Sunnyvale, CA, USA). The kinetic parameters of *At*-ATA for 1-(*R*)-PEA and pyruvate were determined by measuring the activities at different substrate concentrations when either R-MBA or pyruvate was a fixed concentration of 2.5 mM, and the other substrate was changed at 0-3.0 mM until substrate inhibition was observed. The kinetic results were fitted to the Michaelis-Menten equation in Origin 8.0.

### Molecular Dynamics Simulation

We redesigned the 3D structure of *At*-ATA combined with PLP based on the crystal structure of *At*-ATA (PDB ID: 4CE5) from the Protein Data Bank (http://www.rcsb.org). In view of the pretreatment of enzyme without substrate, we had to delete the free Lys180 and PLP-amino donor compound (PDG), and combined PLP with Lys180. According to the experimental conditions, the protonation states of all ionizable residues were assigned based on pKa values from the PROPKA software in combination with visual inspection of local hydrogen bonding networks using Discovery Studio 2018 at pH 8.0, including the protonation status of ionizable residues and lysine affected by the pocket environment.

MD simulation was performed at a constant temperature (313 K) for 50 ns using the Amber 14 force field of YASARA (version 16.4.6) software (http://www.yasara.org). The 3D structures were filled with water with a density of 0.998 mg/L and inserted into a cube with edge lengths of 10 Å. Sodium and chloride ions (0.9%) were added as counter ions to form an electrically neutral system, and the ionizable groups were protonated according to their pKa values at pH 8.0 in the medium. The systems were optimized by three-step energy minimization at the molecular mechanics (MM) level to adjust the poor interatomic interactions. First, the water molecules were minimized while keeping the protein and substrate constrained. Then, the side chains were allowed to relax while the main protein chains were restrained. Finally, the entire system was completely relaxed without any restrictions. After each energy minimization was completed, a 1000-step conjugate gradient iteration loop was performed. Subsequently, the optimized system was gradually heated from 0 to 313 K in a constant volume environment for 150 ps, and then balanced for 150 ps in a constant pressure environment with the density of the system gradually becoming 0.997 g/cm^3^. Finally, a 50 ns MD simulation with a step length of 2.5 fs was completed under constant pressure conditions, and the trajectory was collected every 25 ps. The cutoff value of the van der Waals force and electrostatic interaction during the simulation was handled at 8.0 Å. Analyses of protein structures including root mean square deviation (RMSD) of backbone atom positions, and root mean square fluctuation (RMSF) for individual residues were performed using YASARA (Purmonen et al., 2007; Dong et al., 2018).

## Results

### Selection of Mutants With Increased Thermal Stability *in silico*

**Figure 1:**
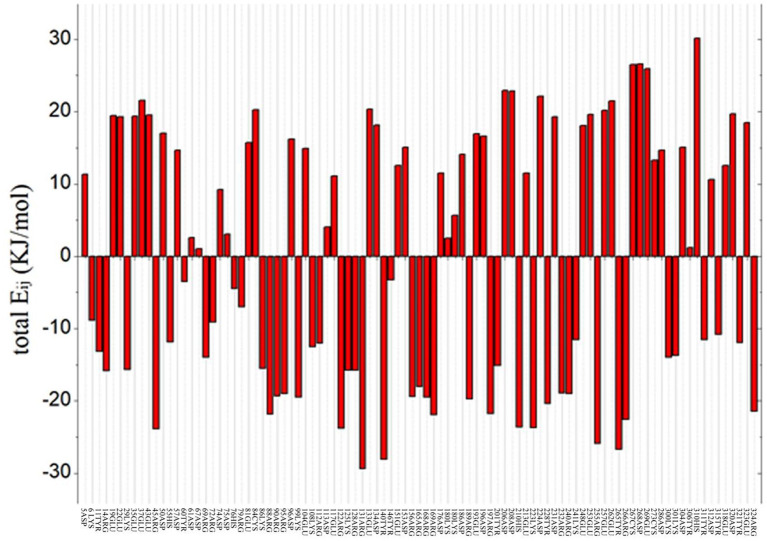
Total energy of each chargeable residue. Positive value indicates the overall contribution of repulsive force to the thermal stability of protein structure, whereas negative value represents the overall contributions from gravitation, which is beneficial to the thermostability of the protein structure.

To predict the residue with an optimized value and analyze the flexibility of the surrounding amino acid residues, ETSS was used to evaluate the interaction of charged amino acids (Zhang et al., 2014). The program was used to calculate the interaction parameters of *At*-ATA, and the total *E*_ij_ shown in Figure 1 reveals that there are 95 charged amino acids in the monomer of *At*-ATA. Thus, we selected amino acids with positive values and far from the active center to mutate to amino acids with opposite or neutral charges. Therefore, four high-value residues located in different loop regions from the surface of the protein were selected (E133, D224, E253, and E262 shown in Supplementary Figure 1).

### Thermostability Analysis of the Mutant *At*-ATA by Alanine Scanning

**Figure 2:**
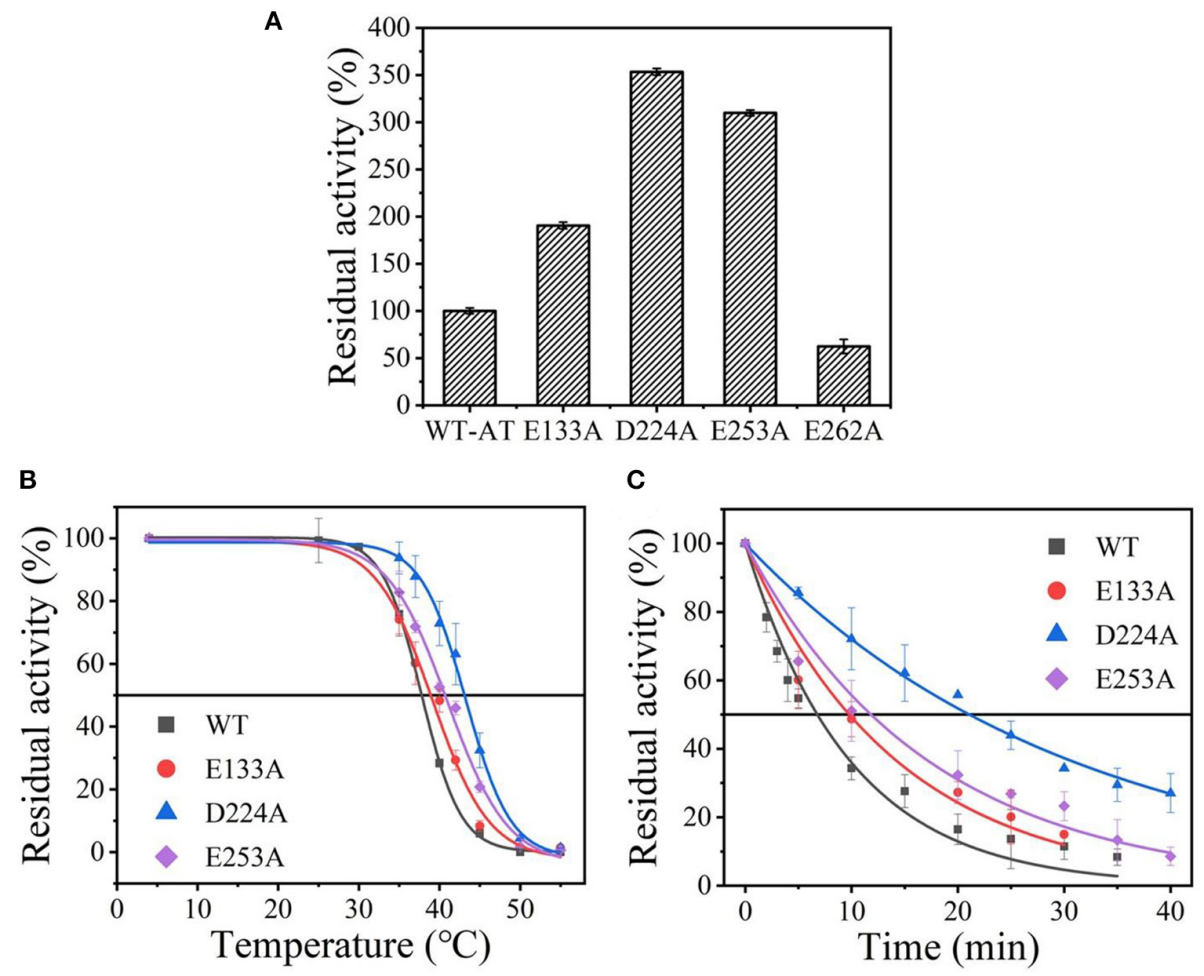
The thermal stability of wild-type *At*-ATA and its mutants by the alanine scanning: **(A)** the enzymatic activity of wild-type *At*-ATA and the four variants, **(B)** the thermal deactivation of wild-type *At*-ATA and the four variants at various temperatures for 10 min ($T_{50}^{10}$), **(C)** the thermal deactivation half-life (*t*_1/2_) of wild-type *At*-ATA and the three variants at 40°C. Black, Wild-type; red, E133A; blue, D224A; and purple, E253A.

Wild-type was scanned for alanine by site-directed mutagenesis and successfully expressed in *E. coli* BL21(DE3). As shown in Supplementary Figure 2, the four purified variants (E133A, D224A, E253A, and E262A) showed a single band with an apparent molecular mass of 35-45 kDa as the wild-type enzyme (36.1 kDa). Except for the mutant E262A, other mutants showed an increased thermostability and activity comparable to that of the wild-type enzyme (data shown in Figure 2A), and its *t*_1/2_ and $T_{50}^{10}$ values were measured. Among them, the D224A mutant showed a significant 3.05-fold increase in *t*_1/2_ at 40°C and withstood a higher temperature value ($T_{50}^{10}$) of 43.1°C, compared to the wild-type enzyme with a *t*_1/2_ of 6.9 min and $T_{50}^{10}$ of 38.5°C (Figures 2B,C and Supplementary Table 2). The results indicate that eliminating the charge of acidic residues on the *At*-ATA surface may improve the thermostability.

### Thermostability Analysis of the Mutant *At*-ATAs by Basic-Amino-Acid Scanning

**Figure 3:**
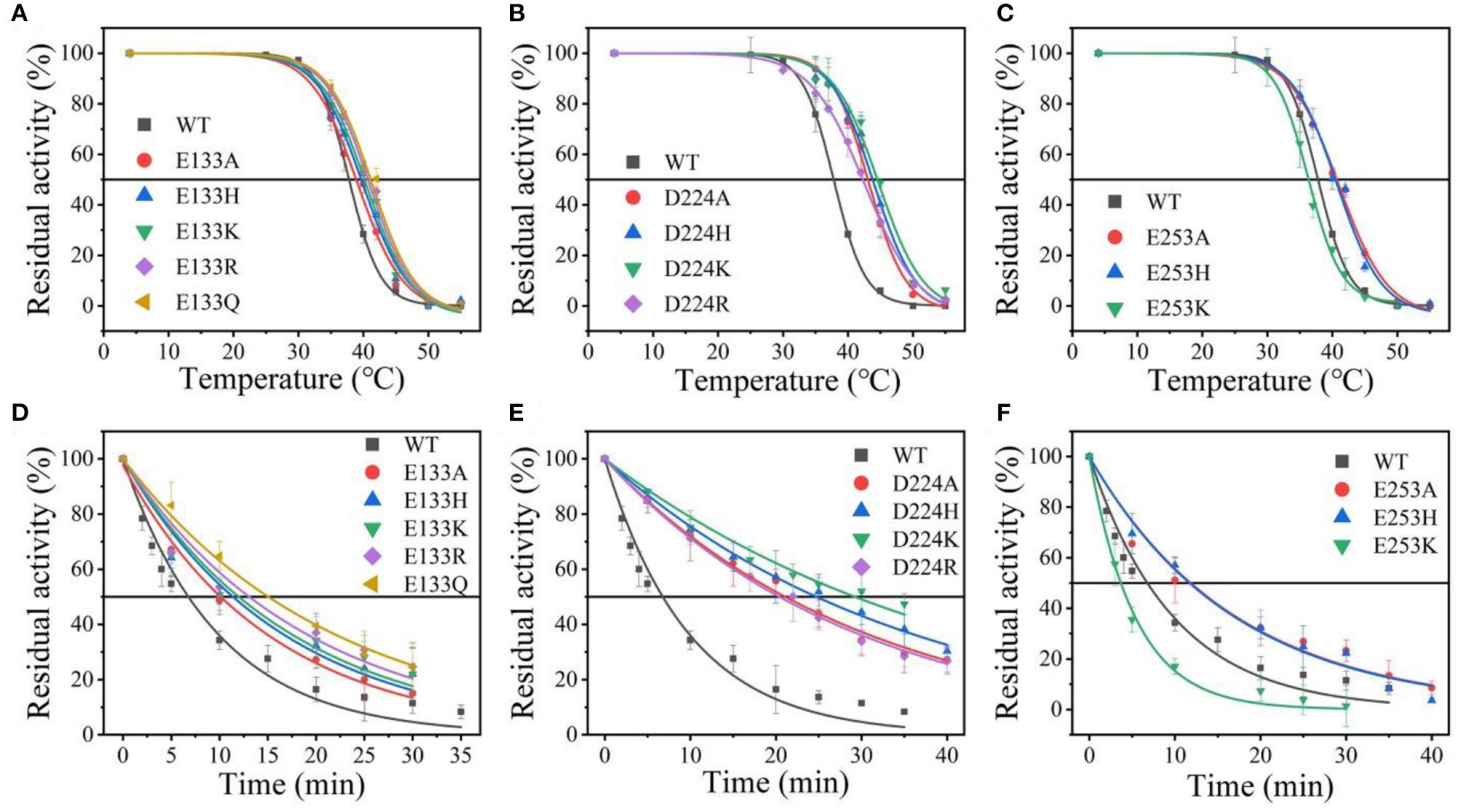
The thermal stability of wild-type *At*-ATA and its mutants in each site. **(A–C)** the thermal deactivation of wild-type *At*-ATA and the 12 variants at various temperatures for 10 min ($T_{50}^{10}$), **(D–F)** the thermal deactivation half-life (*t*_1/2_) of wild-type *At*-ATA and the 12 variants at 40°C.

Suitable basic amino acids were selected as targets, and nine mutants (E133H, E133K, E133R, E133Q, D224H, D224K, D224R, E253H, and E253K) were obtained to analyze the change rule of thermostability affected by the electrostatic interaction combined with the results of alanine scanning. The new mutant consistently showed higher activity at 40°C. Their stabilities were then compared at the preference temperatures. As shown in Figure 3 and Table 1, the $T_{50}^{10}$ values for the E133Q, D224K, and E253A mutants, which is the best one in each mutation group, were 41.12, 44.59, and 40.69°C, respectively, compared with that of the wild-type (38.5°C). The three mutants had consistently higher residual activity at 40°C, which may keep half of the activity 1.71-fold, 2.17-fold, and 4.24-fold higher than the wild-type. The melting profiles of the four enzymes were determined by monitoring the fluorescence of SYPRO Orange dye using DSF over the temperature range of 25–70°C, as a method for enzyme thermostability (Zhu et al., 2019). A melting temperature (*T*_m_) of 47.6 ± 0.2°C was obtained with the fitting data for D224K (Table 1 and Supplementary Figure 3), with *R*^2^ > 0.99 in both cases. The data indicate the consistency of thermodynamic stability.

**Table 1:** The stability of the wild-type and stabilized mutant *At*-ATAs.

**Name**	**$T_{50}^{10}$ (°C)**	***t*_1/2_ (min)**	***T*_m_ (°C)**
WT	38.5 ± 0.5	6.9 ± 0.6	41.3 ± 0.2
E133Q	41.1 ± 0.2	15.0 ± 0.5	42.6 ± 0.2
D224K	44.5 ± 0.2	29.2 ± 0.2	47.6 ± 0.1
E253A	40.7 ± 0.5	11.8 ± 0.6	42.4 ± 0.2

### Kinetics of *At*-ATA and Its Mutants

The *At*-ATA fit the single-substrate Michaelis-Menten kinetics at 25°C when pyruvate or 1-(*R*)-PEA was used as the single variable. Kinetic parameters were measured at 25°C by carrying out 3 min reactions over a range of pyruvate or 1-(*R*)-PEA from 0.125 to 3.0 mM giving the kinetic values shown in Table 2. The $k_{\text{cat}}^{\text{pyruvate}}$ values for E133Q, D224K, and E253A were 2.72-fold, 2.60-fold, and 2.17-fold higher than the wild-type, and for 1-(*R*)-PEA, the $k_{\text{cat}}^{1-\left(\text{R}\right)-PEA}$ values for the E133Q, D224K, and E253A were 1.82-fold, 1.01-fold, and 2.20-fold higher than that of the WT, respectively, showing lower reaction energy requirements for both substrates. The $K_{\text{m}}^{\text{pyruvate}}$ for three mutants were 0.50, 0.35, and 0.69 mM, which were 2.17-fold, 1.52-fold, and 3.00-fold higher than that of the wild-type. In contrast, the $K_{\text{m}}^{1-\left(\text{R}\right)-PEA}$ values for the three mutants were decreased to 0.16, 0.14, and 0.17 mM compared with that of the wild-type (0.23 mM). Thus, the *k*_cat_/*K*_m_ values of the E133Q, D224K, and E253A mutants were 2.74, 3.76, and 2.51 L/(s·mmol) for 1-(*R*)-PEA, and 7.46, 4.64, and 8.38 L/(s·mmol) for pyruvate, respectively.

**Table 2:** Steady-state kinetic constants of wild-type and stabilized mutant *At*-ATAs.

**Name**	$k_{cat}^{pyruvate}$	$K_{m}^{pyruvate}$	***k*_**cat**_/**$K_{m}^{pyruvate}$	$k_{cat}^{1-(R)-\text{PEA}}$	$K_{m}^{1-(R)-\text{PEA}}$	***k*_**cat**_*/***$K_{m}^{1-(R)-\text{PEA}}$
	**(s^**−1**^)**	**(mM)**	**(L/(s·mmol))**	**(s^**−1**^)**	**(mM)**	**(L/(s·mmol))**
WT	0.50 ± 0.01	0.23 ± 0.02	2.22	0.64 ± 0.01	0.23 ± 0.03	2.82
E133Q	1.36 ± 0.03	0.50 ± 0.01	2.74	1.17 ± 0.01	0.16 ± 0.01	7.46
D224K	1.30 ± 0.02	0.35 ± 0.02	3.76	0.65 ± 0.02	0.14 ± 0.01	4.64
E253A	1.73 ± 0.02	0.69 ± 0.01	2.51	1.41 ± 0.01	0.17 ± 0.02	8.38

### Conformation and Energy Change Revealed by Molecular Dynamics Simulation

**Figure 4:**
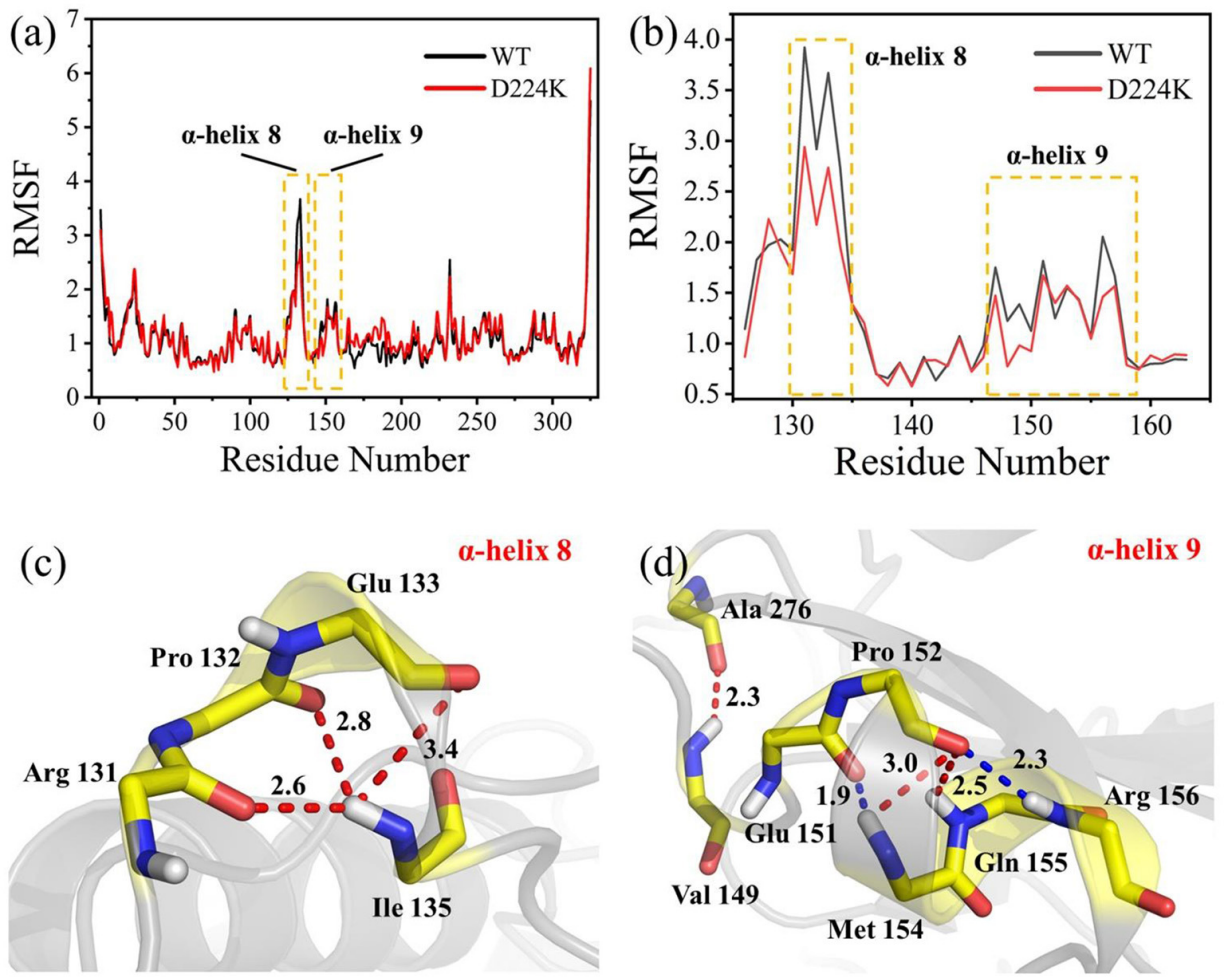
MD analysis of *At*-ATA and D224K using YASARA at 313 K in the last 20 ns. **(a)** The RMSF values of *At*-ATA and D224K; **(b)** the detail RMSF values of α-helix 8 and α-helix 9; **(c,d)** 3D view of two α-helix structures. The hydrogen bonds were displayed by dotted line with red and blue.

**Figure 5:**
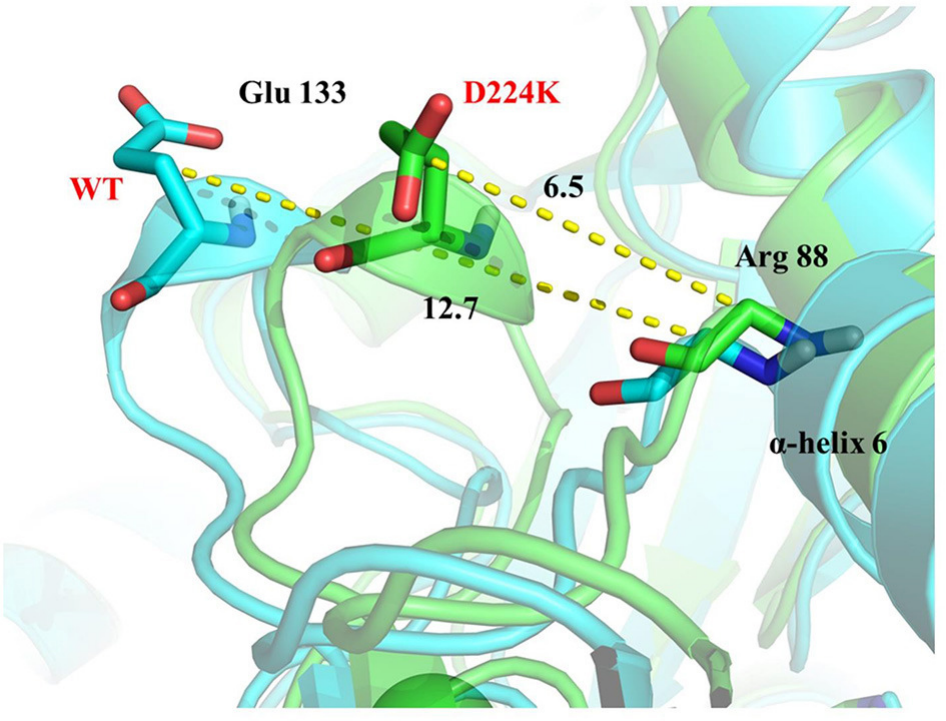
3D view of the surrounding structure of α-helix 8 surrounding on the wild type (blue) and D224K (green).

The stability structure of *At*-ATA and its mutants at 313 K was established by MD simulation and further analyzed. Analysis of the residual level fluctuations demonstrated that the wild-type enzyme is more flexible than mutants at the 130-135 site (belonging to α-helix 8) and 148-158 site (belonging to α-helix 9) (Figures 4a,b and Supplementary Figures 4A,B). In particular, the largest fluctuations at residue Arg131 in the D224K model was 2.81 nm which was 1.09 less than that of the wild-type as simulated using the RMSF. The reason for this increased stability is likely to be the powerful hydrogen bond formed by Ile135 and Arg131, Pro132, and Glu13 from the α-helix 8, which are 2.6, 2.8, and 3.4 Å in length, respectively (Figure 4c). More importantly, these strong hydrogen bonds reduce the distance between α-helix 8 and α-helix 6 as shown in Figure 5, which shorten from 12.7 to 6.5 Å in the D224K model. At the same time, three new hydrogen bond interactions were also added at α-helix 9, as shown in Figure 4d (Ala 276 and Val 149; Pro 152 and Met 154; Pro152 and Gln155 shown in red). In addition, the distance of two hydrogen bonds (the NH in Met154 and the O in Glu151, the NH in Arg156 and the O in Pro152, shown in blue) were reduced to 1.9 and 2.3 Å, respectively, compared with that of the wild-type. These strong hydrogen bonds reduce the flexibility of α-helix 8 and α-helix 9 and greatly stabilize the structure of the protein in the D224K mutant. The RMSF shown in Figures 4a,b reveals that both E133Q and E253A mutants are less flexible than the wild-type, although not as stable as D224K, especially the region at α-helix 8 and α-helix 9. Supplementary Figures 4C–F show four newly added (red) and four shortened (blue) hydrogen bond interactions acting on α-helix 8 and α-helix 9 in the E133Q mutant. For the E253A mutant, five newly added (red) and two shortened (blue) hydrogen bond interactions directly affected the activity of α-helix 8 and α-helix 9, reducing the radius of the helix (Supplementary Figures 4G,H).

## Discussion

Transaminase is a biocatalyst which can efficiently catalyze the synthesis of chiral amines. It has become an important research topic to improve the thermal stability of transaminase by site-directed mutagenesis to expand its application in industry. At present, different protein engineering strategies have been used to improve the thermal stability of transaminases. Among them, some good protein engineering strategies can reliably predict mutants with improved stability, and are easy to implement (Jones et al., 2017). In this work, the results showed that the combination of computer-aided rational design and amino acid scanning proved to be promising for finding mutation candidates with enhanced thermal stability.

In this study, four key residues of *At*-ATA, E133, D244, E253, and E262, were identified to redesign by site-directed mutagenesis combined with the open-source software ETSS. Among them, the alanine scanning results showed that three mutants (E133A, D224A, and E253A) could enhance the thermostability of transaminase. Based on the above results, we screened the most suitable charged amino acids for each site. Finally, E133Q, D224K, and E253A were confirmed to have positive effects on the stability of transaminase. Viewed in PyMOL 2.0.7, Glu133 is located at helix 5, whereas Asp224 belongs to the loop area connected to β-sheet 12 and β-sheet 13, and Glu253 is located at a loop area between helix 8 and β-sheet 12.

To further study the results of the experiment, a virtual mutation model was built by Discovery Studio 2018, and recalculated by YASARA and ETSS. Intermolecular interactions, such as hydrogen bonds, hydrophobic interactions, van der Waals forces, ionic bonds and disulphide bonds play a decisive role in stabilizing the tertiary structure of proteins (Fan et al., 2018; Xie et al., 2019). MD simulation results also support the key role of substitution of positively charged amino acids (glutamine, lysine, histidine, and arginine) or neutral amino acids (alanine) in thermal stability. As shown in Figure 4c, a special “claw structure” was found in D224K mutant, in which Ile135 combined with Arg131, Pro132, and Glu133 three residues. These three hydrogen bonds were formed compared with the wild-type, resulting in the great stability of α-helix 8. The “claw” was connected between α-helix 8 and α-helix 6, thus it would be more inward and closer to the center of the mutant than the wild-type (Figure 5). In addition, the O atom in the main chain of Pro152 formed a hydrogen bond with the N atom in the main chain of Met154, Gln155, and Arg156, which led to the Pro152 firmly catching hold of the center of α-helix 9. Further, a hydrogen bond is formed between Val149 and Ala276 with 2.3 Å, thus approaching the β-sheet 15 and 16 with Ala276 to avoid large fluctuation (Figure 4d). For E133Q, the interactions between Thr146 and Glu155 shortened the distance between α-helix 9 and β-sheet 7 and 16, respectively (Supplementary Figures 4C–F). Similar to the structure of D224K, the strategy of alternating hydrogen bonding reduces the fluctuation range of α-helix 9 in E253A (Supplementary Figures 4G,H). In addition, based on the ETSS analysis, when Asp224 was mutated to Lys224, the *E*_*ij*_ value was decreased from 22.16 to 17.37 kJ/mol in the D224K mutants. For E133Q and E253A mutant, the *E*_*ij*_ values were decreased by 2.17 and 2.28 kJ/mol, respectively, compared with that of the wild-type. This indicates that the entire energy contribution is transferred from an unfavorable to favorable state, which improves the thermal stability significantly. Furthermore, it was found that the effect of oppositely charged residues on the charged amino acids at different positions was different.

## Conclusion

In this study, we obtained three stable mutants, E133Q, D224K, and E253A, according to the ETSS strategy based on the TK-SA algorithm. The mutant D224K not only improved the thermal stability, but also increased the enzyme activity to a certain extent. At the same time, we analyzed hydrogen bond interactions in the two helix regions (α-helix 8 and α-helix 9) by MD simulation, and these were significantly different from the wild type. This study explored the important influence of charge interaction on the structure of transaminase, and provided a feasible strategy for improving the thermal stability of transaminase and promoting its application in industrial production.

## Data Availability Statement

The original contributions presented in the study are included in the article/Supplementary Material, further inquiries can be directed to the corresponding author/s.

## Author Contributions

JH and L-HM designed research and assigned personnel. J-RC and C-JL conducted experiments. F-FF performed the molecular dynamics simulation. H-PW analyzed the data. H-BC provided the technical help. J-RC, C-JL, and F-FF prepared the original draft of the manuscript. YL, SH, and W-RZ reviewed the manuscript and gave suggestions. All authors read and approved the manuscript.

## Conflict of Interest

H-BC was employed by the company Enzymaster (Ningbo) Bio-Engineering. The remaining authors declare that the research was conducted in the absence of any commercial or financial relationships that could be construed as a potential conflict of interest.

## Supplementary Material

Supplementary Figure 1 The 3D structure of *At*-ATA with putative important residues introduced by ETSS and the four residues with strong charge-charge interactions were visualized in the crystal structure of *At*-ATA (PDB ID: 4CE5) using the program PyMOL (http://pymol.org).

Supplementary Figure 2 The SDS-PAGE analysis of wild-type and *At*-ATA mutants. Lanes: 1, Protein marker; 2, Wild-type; 3, E133A; 4, D224A; 5, E253A; 6, E262A.

Supplementary Figure 3 Thermal unfolding of wild-type *At*-ATA and its mutants were monitored by DSF.

Supplementary Figure 4 MD analysis of *At*-ATA and E133Q and E253A using YASARA at 313 K in the last 20 ns. **(A)** The RMSF values of *At*-ATA and its mutants. **(B)** The detail RMSF values of α-helix 8 and α-helix. **(C–F)** The hydrogen bond interaction in two α-helix for the mutant E133Q; **(G,H)** the hydrogen bond interaction in two α-helix for the mutant E253A; The added bonds were displayed by red, and the blue one showed a shortened change compared with the wild type.

Supplementary Table 1 Primers used for site-directed mutagenesis.

Supplementary Table 2 The stability of the wild-type and stabilized mutant *At*-ATAs.
