# Supplementary figures and images for: Improving the Thermostability and Activity of Transaminase From Aspergillus terreus by Charge-Charge Interaction
Source: Front Chem. 2021 Apr 14;9:664156. doi: 10.3389/fchem.2021.664156 (PMC8081293; doi:10.3389/fchem.2021.664156)

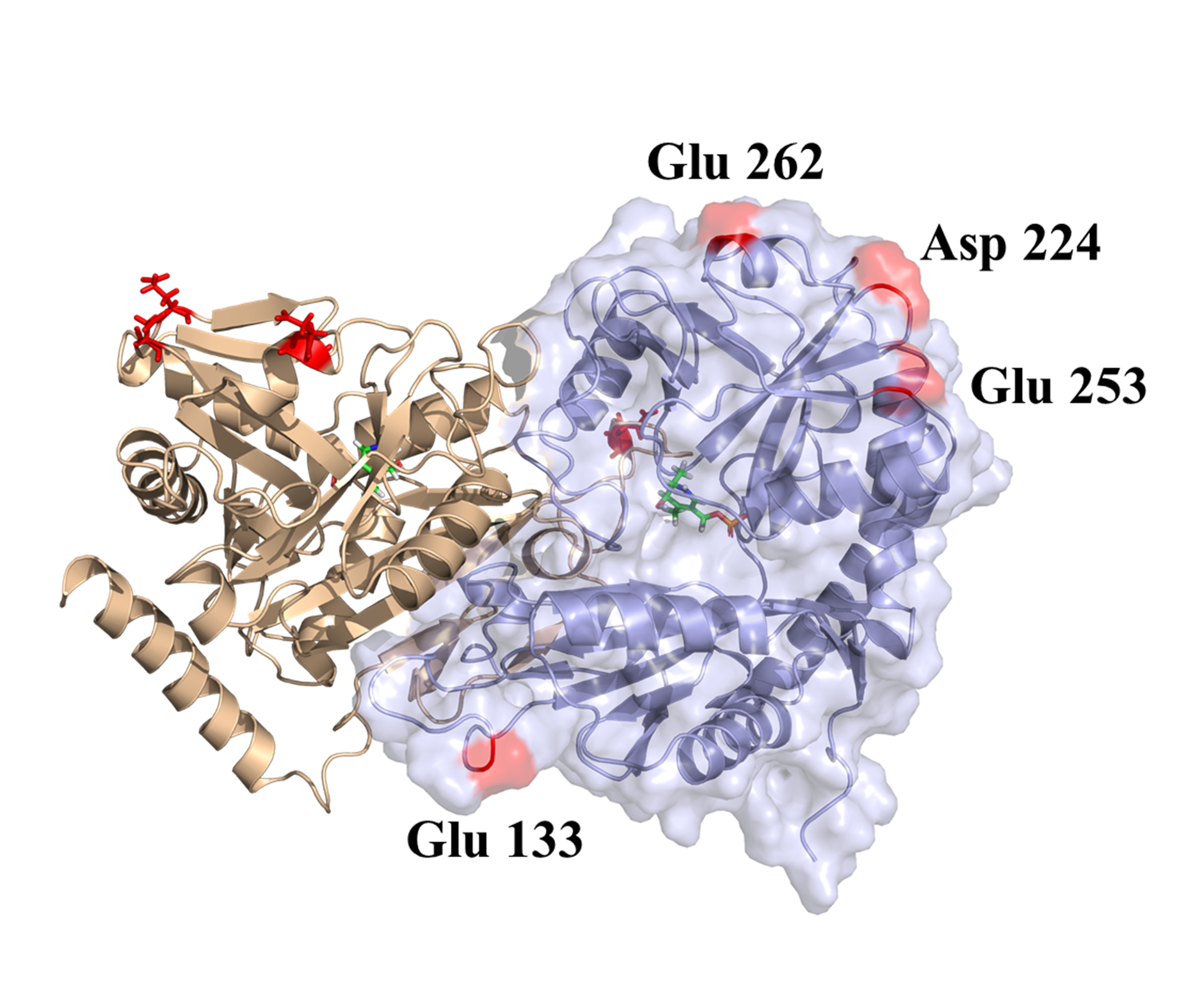

Supplement: Supplementary Figure 1 — The 3D structure of At-ATA with putative important residues introduced by ETSS and the four residues with strong charge-charge interactions were visualized in the crystal structure of At-ATA (PDB ID: 4CE5) using the program PyMOL (http://pymol.org). [file Image_1.JPEG]

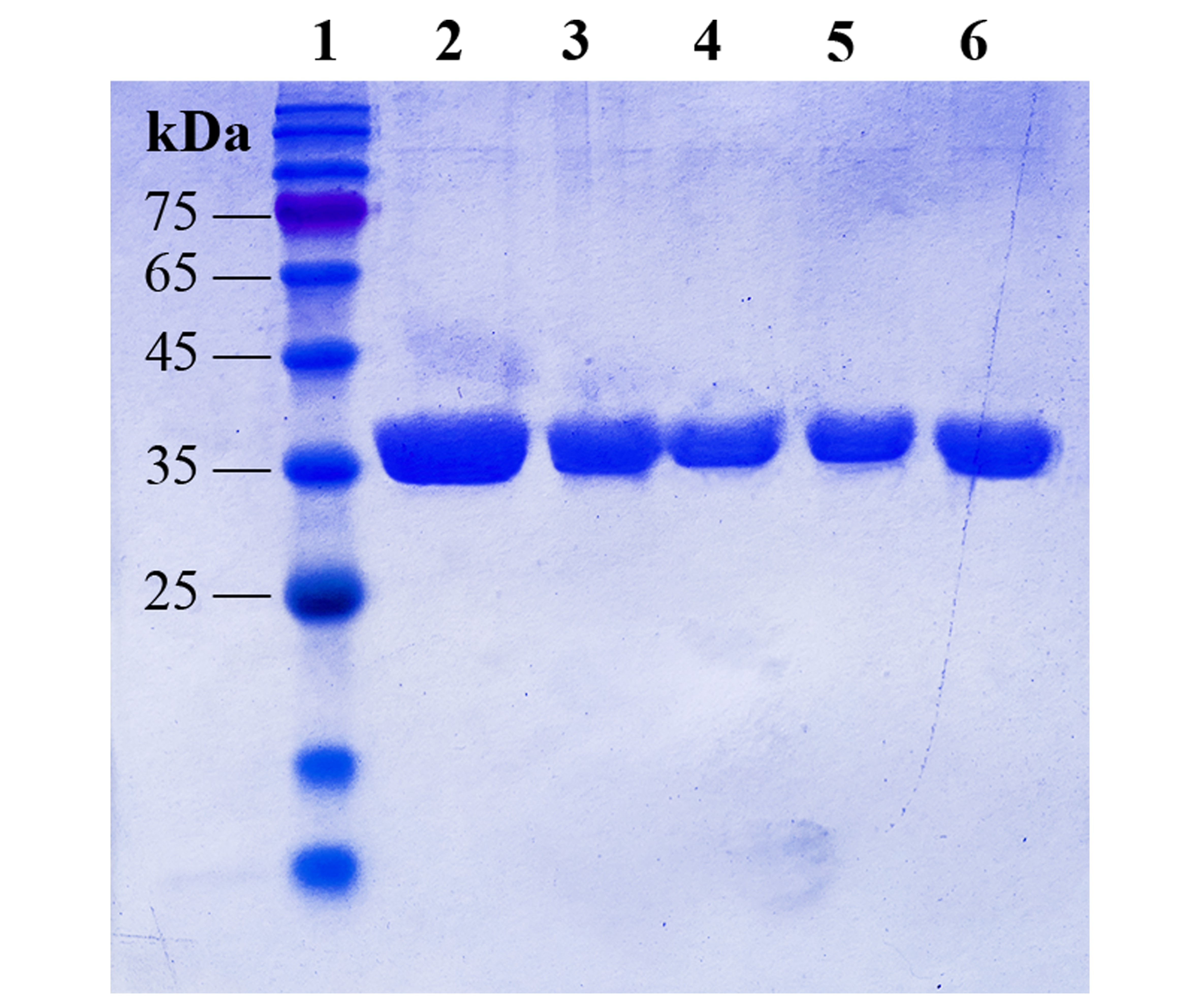

Supplement: Supplementary Figure 2 — The SDS-PAGE analysis of wild-type and At-ATA mutants. Lanes: 1, Protein marker; 2, Wild-type; 3, E133A; 4, D224A; 5, E253A; 6, E262A. [file Image_2.JPEG]

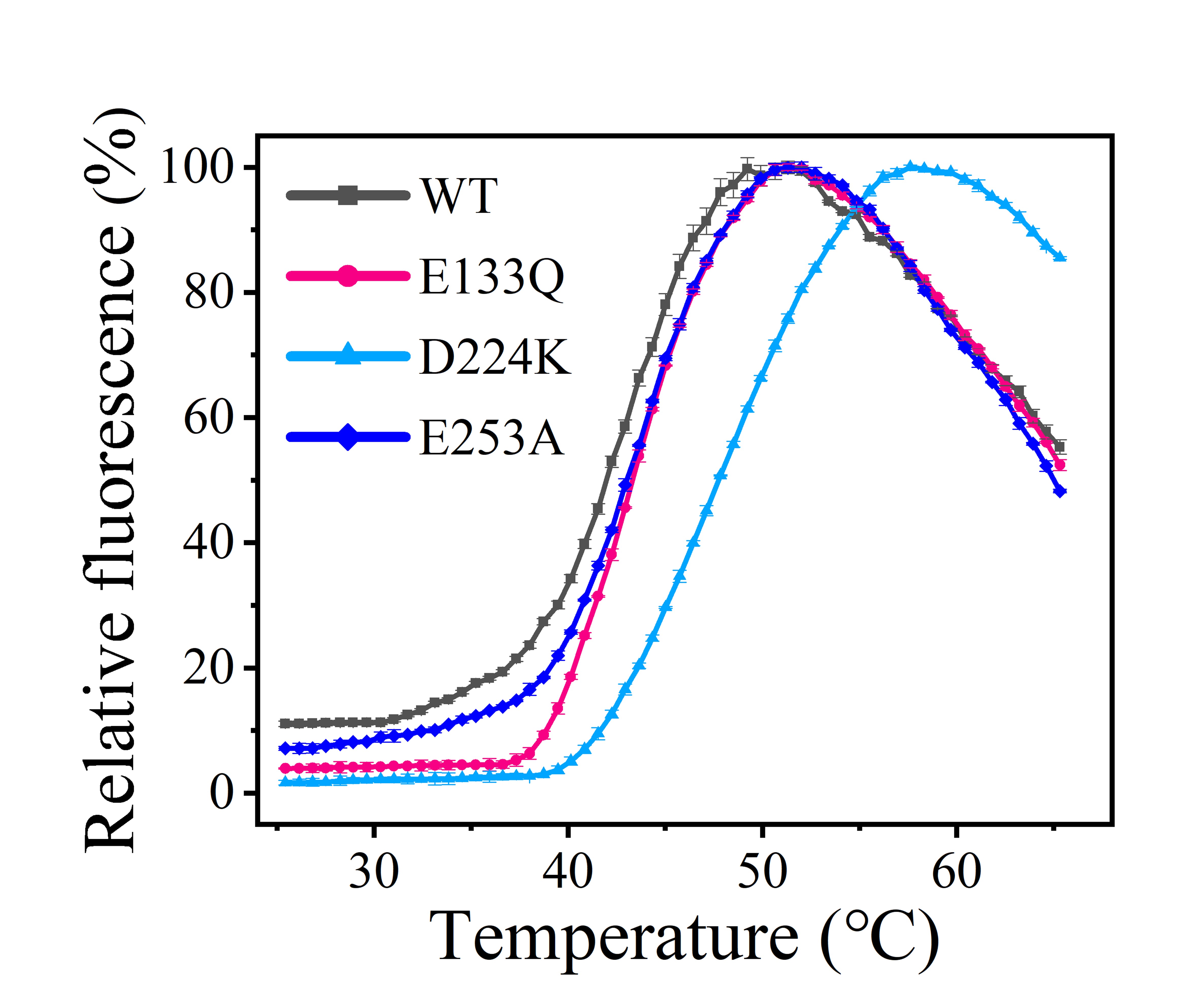

Supplement: Supplementary Figure 3 — Thermal unfolding of wild-type At-ATA and its mutants were monitored by DSF. [file Image_3.JPEG]

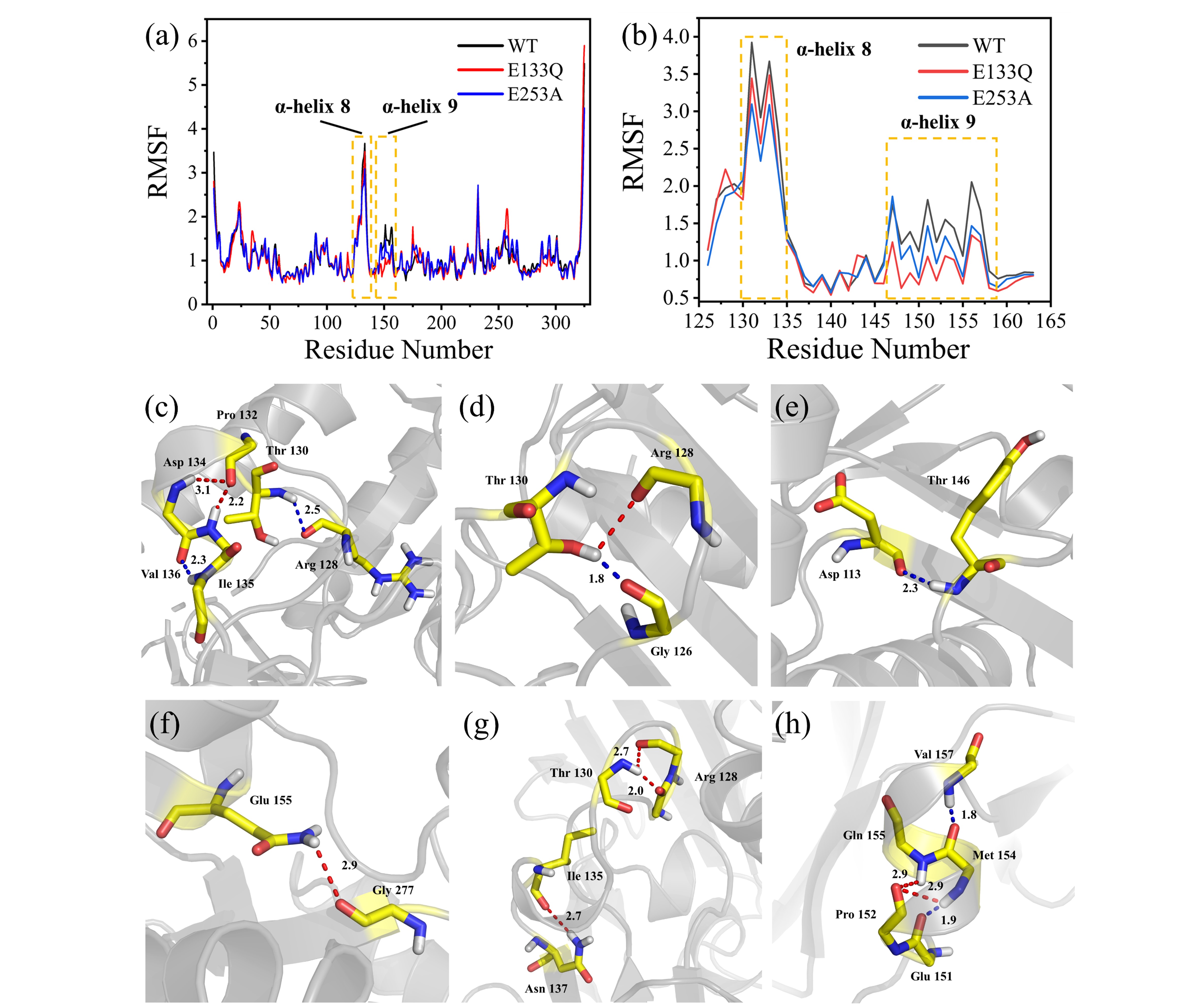

Supplement: Supplementary Figure 4 — MD analysis of At-ATA and E133Q and E253A using YASARA at 313 K in the last 20 ns. (A) The RMSF values of At-ATA and its mutants. (B) The detail RMSF values of α-helix 8 and α-helix. (C–F) The hydrogen bond interaction in two α-helix for the mutant E133Q; (G,H) the hydrogen bond interaction in two α-helix for the mutant E253A; The added bonds were displayed by red, and the blue one showed a shortened change compared with the wild type. [file Image_4.JPEG]

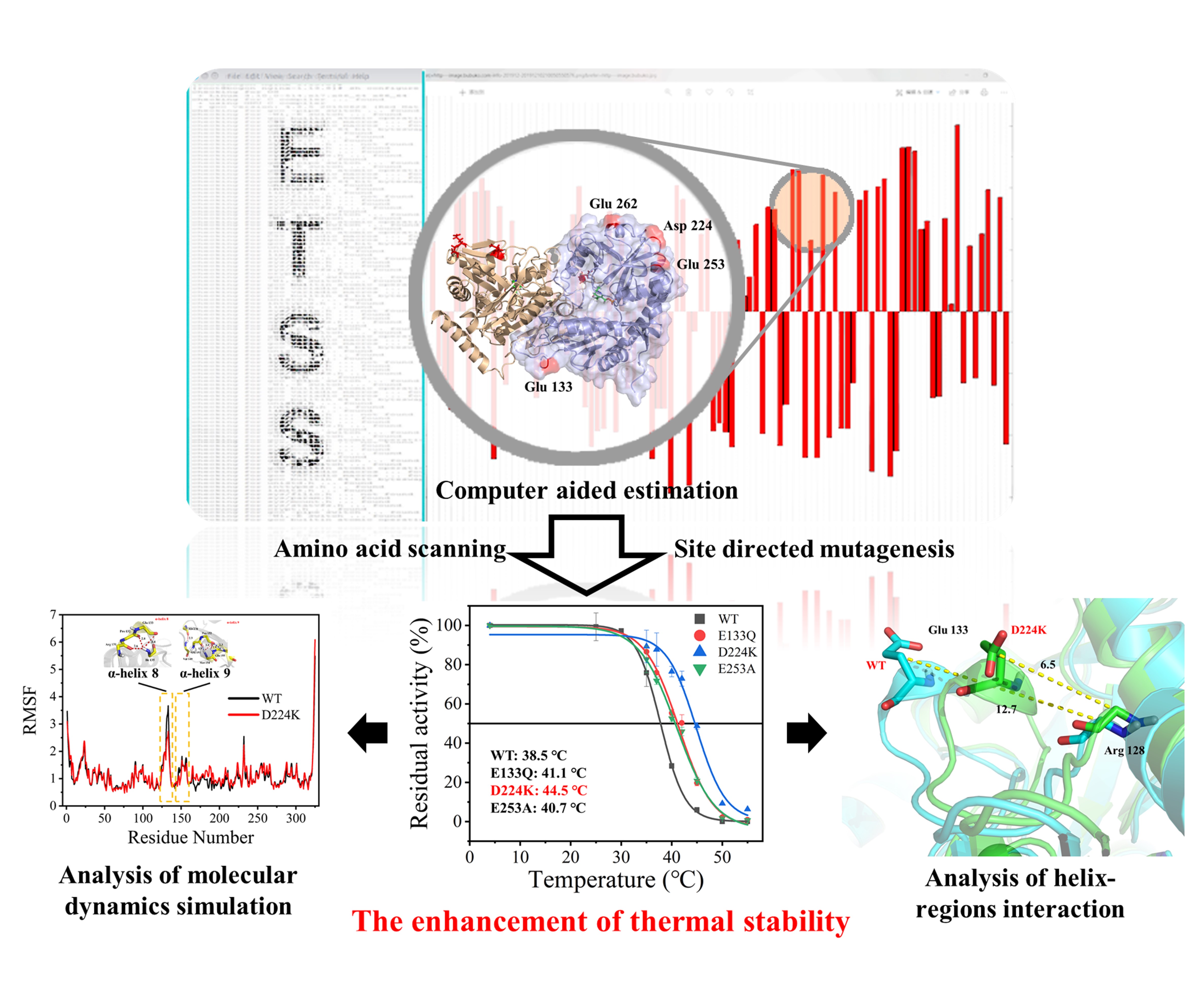

Supplement: Supplementary file 7 [file Image_5.JPEG]
